# Opportunism or aquatic specialization? Evidence of freshwater fish exploitation at Ohalo II- A waterlogged Upper Paleolithic site

**DOI:** 10.1371/journal.pone.0198747

**Published:** 2018-06-18

**Authors:** Irit Zohar, Tamar Dayan, Menachem Goren, Dani Nadel, Israel Hershkovitz

**Affiliations:** 1 Beit Margolin Biological Collections, Oranim Academic College, Kiryat Tivon, Israel; 2 Zinman Institute of Archaeology, University of Haifa, Mount Carmel, Haifa, Israel; 3 School of Zoology, Tel Aviv University, Tel Aviv, Israel; 4 The Steinhardt Museum of Natural History, Tel Aviv University, Tel Aviv, Israel; 5 Department of Anatomy and Anthropology, Sackler Faculty of Medicine, Tel Aviv University, Tel Aviv, Israel; 6 The Dan David Center for Human Evolution and Biohistory Research, Tel Aviv University, Tel Aviv, Israel; 7 The Shmunis Family Anthropology Institute, Tel Aviv University, Tel Aviv, Israel; Institucio Catalana de Recerca i Estudis Avancats, SPAIN

## Abstract

Analysis of ca. 17,000 fish remains recovered from the late Upper Paleolithic/early Epi-Paleolithic (LGM; 23,000 BP) waterlogged site of Ohalo II (Rift Valley, Israel) provides new insights into the role of wetland habitats and the fish inhabiting them during the evolution of economic strategies prior to the agricultural evolution. Of the current 19 native fish species in Lake Kinneret (Sea of Galilee), eight species were identified at Ohalo II, belonging to two freshwater families: Cyprinidae (carps) and Cichlidae (St. Peter fish). Employing a large set of quantitative and qualitative criteria (NISP, species richness, diversity, skeletal element representation, fragmentation, color, spatial distribution, etc.), we demonstrate that the inhabitants of Ohalo II used their knowledge of the breeding behavior of different species of fish, for year-round intensive exploitation.

## Introduction

The contribution of small game species to the human diet is recognized since the Middle Paleolithic [1–7]. It is therefore surprising that the contribution of fish has generally been ignored, as in terms of food distribution, diversification, intensification, dietary costs, and benefits, the aquatic fauna presents a high diversity of abundant and easily collected food that in many cases does not require specialization and produces high return rates [8–11]. The notion that “…*the use of fish in the Middle Paleolithic was*, *at best*, *very scanty*” [12]; p.335), has led to the incorrect conclusion that fish exploitation became a major activity only during the Upper Paleolithic, mainly towards the Terminal Pleistocene and early Holocene (ca. 12 ka BP). Two major causes have been suggested for the intensified exploitation of aquatic resources: *1*) a decrease in hunting options for coastal groups; and *2*) the need for alternative sources of proteins and calories [12]. This is questionable, however, as unlike marine resources, there is evidence to suggest that freshwater resources had been exploited since the Early Pleistocene, i.e., 1.95 mya [1, 13–17].

In the current study we demonstrate that the intensive exploitation of freshwater habitats, and the fish inhabiting them, was practiced in parallel to terrestrial animal exploitation long before the Neolithic revolution; that engaging in fishing was a year-round activity and not an opportunistic one; and that the nutritional value of fish was high, while the energetic costs of obtaining it were low.

**Fig 1:**
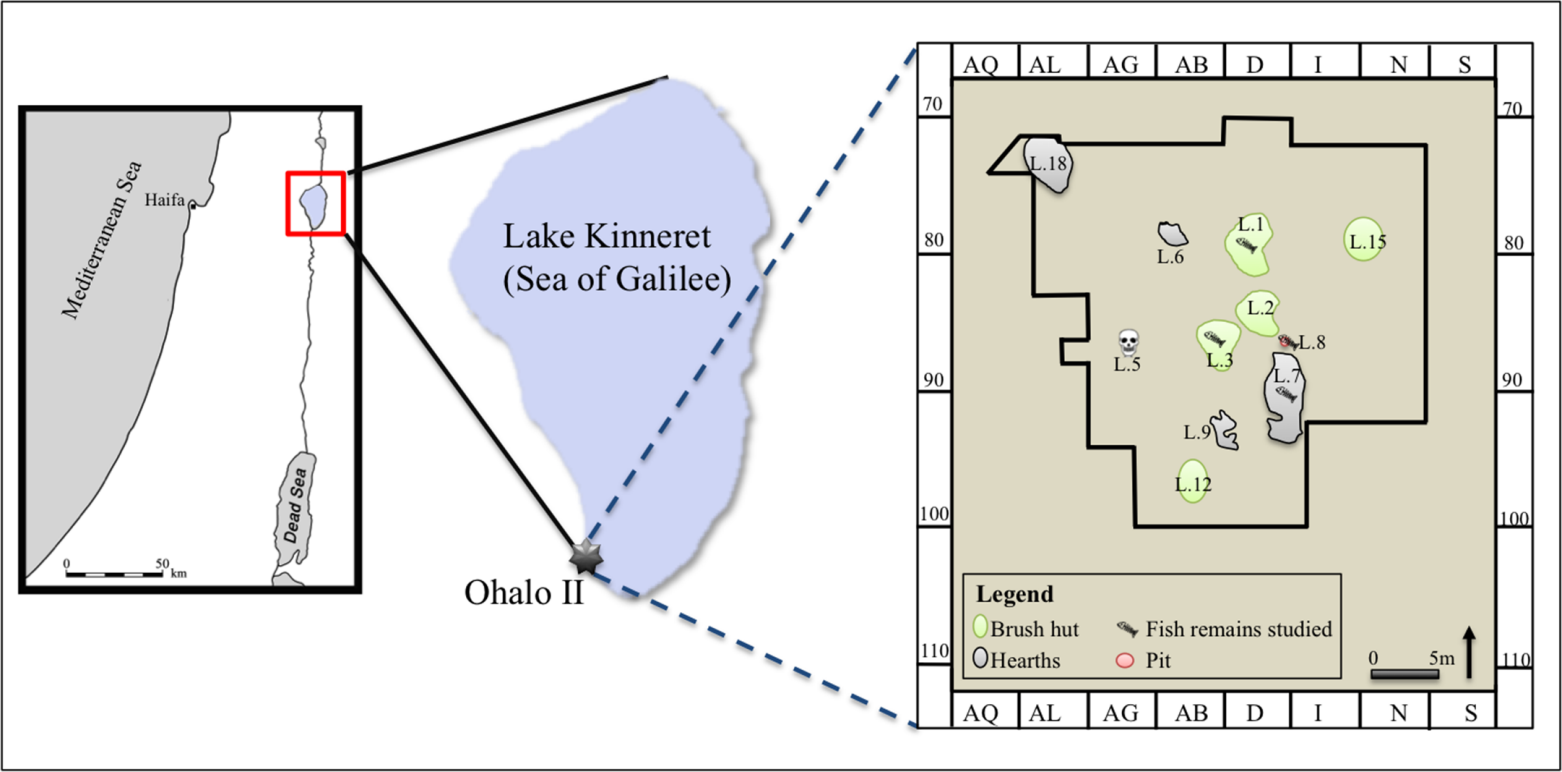
Map of Israel, location of the Ohalo II site, and the excavated loci from which fish remains were examined for this study.

An exceptional opportunity to investigate the role of fish in the Late Upper Paleolithic (LUP) hunter-gatherer economy is provided by the submerged site of Ohalo II (^14^C 20–23,000 calBP), which was occasionally exposed on the southern shore of Lake Kinneret between 1989–2001 (Fig 1) [18–20]. In addition to the diverse and well-preserved botanical and faunal remains (due to the anaerobic conditions) recovered at the site, thousands of fish bones were recovered embedded within the cultural layers [21–27].

In this study we examined the role of Lake Kinneret wetland habitats and their fish communities in the late Upper Paleolithic subsistence economy. The exceptionally large assemblage of well-preserved fish remains from Ohalo II enabled us to examine the association between the fish assemblage’s characteristics (species richness, diversity, skeletal element representation, and fragmentation, etc.) and human activities at the site (dietary preferences, fishing grounds, techniques, seasonality, and processing methods).

## Background

### Lake Kinneret

Lake Kinneret (Fig 1) is situated at the northern part of the Jordan Rift valley, in the north of Israel (GPS: Latitude: 32° 49' 59.99" N; Longitude: 35° 34' 59.99" E). The modern lake evolved during the Late Pleistocene from ancient water bodies that filled the Kinneret tectonic depression [28–31]. Today, the lake's water level is ca. 209 m below sea level, and it is 22 km long (N-S), 12 km wide (S-W), and up to 43 meter deep [32]. At present, the lake is mainly fed from the north by the Jordan River, which also drains the lake southwards. The lake is warm and monomictic, and its water level fluctuates up to 4 m depending on precipitation, evaporation, and water abstraction [33].

### Ohalo II site

Ohalo II is a late Upper Paleolithic (locally termed Early Epipaleolithic) submerged site, located on the south-western shore of Lake Kinneret (GPS: 32°43'18.12" N 35°34'10.37" E), at 212–213 meters below sea level (Fig 1) [20, 34]. The site is estimated to cover an area of 2,000 m^2^, of which 400 m^2^ have been excavated during several seasons (1989–1991; 1999–2001). Several *in-situ* features were exposed (Fig 1), including the floors of six brush huts, open-air hearths, and other installations [22, 35–37]. Brush hut 1 (Locus 1) is exceptional in terms of its size and excellent preservation of charred wall bases made of salt cedar (*Tamarix*) and oak (*Quercus*), three successive floors with activity areas, grass bedding, an *in-situ* grinding stone for cereal processing, and fragments of small twisted fibers [22, 25, 27, 36–40].

Additionally, the remains of three individuals were recovered at Ohalo II site: a mandible (Ohalo II, H-1:); a complete articulated skeleton buried in a flexed position (Ohalo-II, H-2); and two humeri (Ohalo II, H-3) [35]. Diverse faunal remains were found, including medium and small-sized mammals, reptiles, birds, mollusks, and thousands of fish remains [15, 17, 21–23, 26, 41, 42]. A wide diversity of edible seeds and fruits were also recovered and more than 150 plant taxa have been identified [25, 27, 40, 43].

The site has been dated to 22,500–23,500 calBP, based on 50 ^14^C dates of in-situ botanical remains (11 loci were directly dated by 34 ^14^C samples), including samples from pre-occupation and post-occupation layers [20, 22, 27, 34, 36, 37, 44, 45]. These dates falls within the range of the Late Upper Paleolithic, as further supported by the lithic assemblages (tools and manufacture debris) [46]. Altough each of the loci examined may represent different stage of occupation, the uniformity of the flint assemblage suggest that Ohalo II was repeatedly occupied by the same cultural entity, with a short gap of few years between them [34].

### Lake Kinneret native ichthyofauna

The present ichthyofauna of Lake Kinneret comprises 19 indigenous fish species (Table 1) from six families that originate from Africa, Central Asia, and Europe [47–53]. These include several endemic species: *Mirogrex terraesanctae* (Kinneret bleak, “Lavnun Ha'Kinneret", previously assigned to *Acanthobrama*; Cyprinidae), *Tristramella simonis simonis* (“Tvarnun simon,” Cichlidae), *Tristramella sacra* (“Long jaw Tvarnun listani”, Cichlidae), and *Astatotilapia flavijosephi* (Josephus cichlid, "Amununit Yosef”, Cichlidae) [48, 50, 54]. Commercial fishing reports from 1936 indicate that the most abundant captured fish at that time were: *M*. *terraesanctae* (36%), *Luciobarbus longiceps* and *Carasobarbus canis* (33%), and Cichlidae (31%) [55].

**Table 1:** List of Lake Kinneret and Jordan Rift valley fish, their maximum total length (TL†), presence at Ohalo II, season of breeding (winter in green and spring-summer in red), and breeding area [49, 52, 53, 83].

				Breeding				Season										
		Max. TL	Ohalo-II	Autumn				Winter			Spring				Summer			Breeding area
Family	Species	(mm)	Presence	9	10	11	12		1	2	3	4	5	6		7	8	
**CYPRINIDAE**	*Acanthobrama lissneri*	**130**	-															Rivers, on stones and algae
	Mirogrex terraesanctae*	**150**	+															Shallow water, on stones
	*Carasobarbus canis*	650	+															Shallow water on gravel.
	*Luciobarbus longiceps*	750	+															Shallow water on gravel.
	*Capoeta damascina*	450	+															Shallow water, on gravel.
	*Garra jordanica*	**120**	-															On stones
	*Hemigrammocapoeta nana*	**120**	-															Shallow water
	*Pseudophoxinus kervillei*	**100**	-															Shallow water, on stones.
**NEMACHEILIDAE**	*Oxynoemacheilus leontinae*	**73**	-															Shallow water, on stones.
	*Oxynoemacheilus jordanicus*	**120**	-															Shallow water, on stones and roots.
**CLARIIDAE**	*Clarias gariepinus*	1500	-															Shallow swampy water
**CYPRINODONTIDAE**	*Aphanius mento*	**50**	-															Shallow water, in vegetation.
**CICHLIDAE**	*Coptodon zillii*	300	+															Shallow water, vegetated areas, substrate breeder
	*Oreochromis aureus*	350	+															Min temp 20°C. Shallow weedy area, substrate breeder, female carry fertilized eggs to pelagic zone
	*Sarotherodon galilaeus*	380	+															Shallow littoral, mouth breeder.
	*Haplochromis flaviijosephi*	**130**	-															Shallow sandy water, substrate and mouth breeder
	Tristramella sacra*	300	+															Shallow sandy water, substrate and mouth breeder
	Tristramella simonis simonis*	250	?															Shallow sandy water, substrate and mouth breeder
**BLENIIDAE**	*Salaria Fluviatilis*	**150**	-															Shallow water under the stones

* Lake Kinneret endemic species

† Fish with maximum total Length (TL) shorter than 220 mm are marked with gray background color.

## Material

### The fish bone assemblage

The studied sample of fish bones (ca. 17,000; S1 Table) was recovered from four loci: Loci 1 and 3 (brush hut floors), Locus 7 (an open-air activity area with hearth remains), and Locus 8 (a small pit) (Fig 1). Sediments were wet sieved through a 1 mm mesh and fish remains were analyzed and identified in the lab under a Zeiss Stereomicroscope (Zeiss Stemi DV4). Each bone received a catalogue number (S1 Table), and the assemblage is stored at the National Natural History Collections of the Hebrew University of Jerusalem.

Terminology and calculations were performed following standard zooarchaeological approaches and methods [56–62]. The number of identified specimens (NISP) was used as a basic quantitative unit for measuring fish taxonomic presence, ordinal ranking, relative abundance, body part representation, and spatial distribution [59, 63, 64].

As no simple procedure exists to distinguish cultural from biological aquatic accumulations, we employed various parameters that assist in distinguishing anthropogenic from non-anthropogenic accumulations [16, 65–74]. The archeological context of the fish remains was pivotal in selecting the samples for the current analysis and evaluating their cultural role in Ohalo II.

### Fish composition and diversity

Since the structure of biological communities tends to change through time, identification of the taxonomic composition and diversity of a fossil assemblage is challenging [75, 76]. Ohalo II fish remains were identified according to several reference collections of modern and fossil fish. These comprised a native fish collected from Lake Kinneret, Lake Hula, the Jordan River, and the coastal rivers of Israel [16, 42, 77], and modern and fossil reference collections from the Levant and Africa, housed at the Natural History Museums of Brussels and London.

Taxonomic abundance was measured as percentage of the total NISP [63, 64]. Species richness (S') was calculated according to the number of genera identified [78]. Species diversity was calculated by using Shannon-Wiener diversity (H') and Brillouin Index (HB) [59, 78, 79]. The Shannon-Wiener index was used since it is more sensitive to the less abundant species and to species richness as a whole. However, since this index assumes a random sample, we also calculated the Brillouin Index, which does not require random sampling.

We used Sørensen similarity index (Krebs 1999) to compare species diversity from three assemblages: 1). Ohalo II; 2) Lake Kinneret littoral ichthyofauna [52, 74, 80]; and 3). Lake Kinneret natural death assemblage [74]. The Sørensen similarity index formula is (Krebs 1999): S_S_ = 2a/(2a + b + c), where a = number of species common to both sites; b = number of species unique to the first site; c = number of species unique to the second site (S_S_ is usually multiplied by 100%).

As fish composition and diversity are prone to bias depending on sample size, we used the rarefaction technique to examine the influence of sample size on species richness and bone representation at Ohalo II, by loci (Analytic Rarefaction *V*.*1*.*3*, developed for Mac: [81]. Rarefaction calculates the expected number of species in larger samples of *n* individuals [82].

#### Ethics statement

The fish collected for this study were purchased from the local fish market. No permits were required for the described study.

### Bone representation and preservation

#### Skeletal element representation

Relative bone representation (RBR) was calculated for each taxon from the NISP values of individual skeletal elements. We also calculated the RBR when all identified bones of a given taxa were grouped into nine cranial and postcranial anatomic regions: neurocranium, branchial region, hyoid region, oromandibular region and opercular series from the cranial region, and appendicular skeleton, median fins, Weberian apparatus and vertebral column from the postcranial region [62, 74, 84, 85].

The survival index (SI) was calculated as the ratio between the numbers of observed bones (NISP) and the numbers of expected bones (per skeletal element and per anatomic region). The expected bones representation was calculated (per taxon) as the proportion of a given bone (e.g., vertebrae) in a complete fish skeleton multiplied by the total NISP (regardless of % fragmentation) [86]. When SI = 1, the observed NISP equals the expected NISP. SI >1 implies over-representation, while SI < 1 indicates under-representation at the site. The differences observed in the numbers of observed and expected bones (NISP) were compared using chi-square contingency tests, with df = n-1. We further examined whether the obtained SI values were affected by the bones’ state of fragmentation (as described below).

#### Fragmentation

Since a high NISP may result from a high degree of fragmentation, for each bone we also evaluated the relative percentage of preservation [86]. For further analysis and comparison between assemblages, we classified the bones’ rate of fragmentation into three groups: "highly fragmented"–less than 50% of the bone preserved; "fragmented"– 51–75% of the bone preserved; and "well preserved” > 76% of the bone preserved.

Burning: Changes in bone color are commonly used for identifying burnt bones [68, 87]. However, at waterlogged sites bone color can also change due to mineral staining [88, 89]. To distinguish between the two (burning vs. mineral stained bones), we selected representative bones for the various colors (brown, dark brown, black, gray, white) and examined their mineralogical composition with a Fourier Transform Infra-red (FTIR) spectrometer (from MIDAC Corporation, Costa Mesa, CA, USA, housed at the Weizmann Institute, Rehovot, Israel) [90, 91].

#### Bone spatial distribution

Bone spatial distribution patterns (clumped, random, or uniform) were calculated for loci with NISP > 900, using the standardized Morisita index of dispersion [78]. In addition, we calculated the standardized scatter frequency (BSF) as the number of bones (NISP) in each excavated unit divided by the volume of that unit (0.5 x 0.5 x 0.05 m) [64, 74, 86, 92, 93].

### Fish economic value

#### Differentiation between large and small cyprinid

Differentiations between large and small cyprinids was carried out based on vertebrae centrum dimensions: maximum width, length and height [94, 95]). Width diameter <3.5mm< was used to differentiate small from large cyprinids (TL <220mm<) [42]. Vertebrae larger than 3.5 mm in width can represent only three taxonomic groups: *Luciobarbus longiceps*, *Carasobarbus canis*, and *Capoeta damascina*; whereas smaller vertebrae may represent the full taxonomic composition of cyprinids. Additionally, we used predicting equations for body size (length, body mass), for each species separately, based on the atlas and axis vertebrae dimensions [42].

#### Fish exploitation index (prey choice model)

Fish exploitation index (FEI) was calculated based on optimal foraging and prey choice models [9, 96]. These models are based on the relative abundance index (AI), assuming that the best resources collected are those that provide the highest energetic return and demand the lowest energetic costs [97, 98]. Given the relationship between body size, habitat, fishing methods, technology at the time, and resource ranking, we regarded large littoral fish as ‘high-ranked’ and small pelagic fish as ‘low-ranked’. We estimated changes in the contribution of high and low-ranked resources to the human diet, following Butler’s FEI formula [9]. When the index value is close to 1, the contribution of large, higher-ranked fish is high.

At Ohalo II, large taxa refer to fish with maximum total length (TL) greater than 220 mm (Table 1). Cyprinids comprised the following species: *Carasobarbus canis*, *Luciobarbus longiceps*, and *Capoeta damascina*; and cichlids comprised: *Coptodon zillii*, *Oreochromis aureus*, *Sarotherodon galilaeus*, *Tristramella sacra*, and *Tristramella simonis simonis*.

### Validity: Are fish bone accumulations at Ohalo II anthropogenic?

Distinguishing between fish bone accumulation due to anthropogenic activities and post-depositional processes (following natural death) at submerged archaeological sites is critical [16, 66, 74, 84, 85, 99–102]. Since Ohalo II is a waterlogged site, we cannot disregard the possibility that the fish assemblages may have resulted from natural death. To eliminate such possibility, we compared the Ohalo-II fish assemblages with a natural death assemblage, sampled from an area located 150 m north of Ohalo-II site [74]. The area selected (100 X 50 m) was divided into squares of 0.5 X 0.5 m. Using a random sampling program, 24 squares were selected for excavation [74]. Each square was excavated to a maximum depth of 30-50cm, according to its lithological composition. The bottom clay layer was radiocarbon dated to 1515 ± 50 y BP (uncalibrated ^14^C and uncorrected to reservoir age, University of Arizona, Tucson). A total of 5,795 fish remains from the 24 random squares, was recovered and studied [74].

For comparison among the assemblages, based on several studies, we established a “diagnostic signature criteria” [8, 16, 65–67, 71, 73, 74, 84, 85, 103–106]. Moreover, the fish taxon representation at Ohalo II (relative distribution of the four taxonomic groups in each locus) was compared with the taxonomic composition of the lake’s littoral zone and natural assemblage [74], using a multivariate procedure of multidimensional scaling (MDS). MDS is used to provide a visual representation of the pattern of loci proximities (including natural accumulation) in regard to taxon presentation. The MDS is an ordination procedure that compresses multidimensional space onto a simple two-dimensional representation (Borg, 1981). It has no underlying assumptions about the normality or linearity of the data. The fit to the two-dimensional model is evaluated by a stress factor, which ideally should be lower than 0.1. MDS plots the variables on a map that places similar variables adjacent to each other while those that greatly differ are located at a greater distance.

## Results

### Fish composition and diversity

The taxonomic composition was calculated from a sample of 16,939 fish remains (NISP) recovered from Loci 1, 3, 7, and 8 (Table 2). Of the six native families that currently inhabit Lake Kinneret (Cyprinidae, Cichlidae, Nemacheilidae, Clariidae, Cyprinodontidae, Blenniidae), only cyprinids and cichlids were identified at Ohalo II. Identification to genus and species level was possible for 28% of the bones (NISP = 4,746; Table 2). Of the 19 native extant fish species in Lake Kinneret, eight species were identified at Ohalo II (Tables 1 and 2). These include four of the seven native taxa of cichlids, including the endemic genus *Tristramella* sp. For Cyprinidae, of the nine native species, four were identified at Ohalo II, including the endemic species *Mirogrex* (*Acanthobrama*) *terraesanctae* (Tables 1 and 2). The similarity (Sørensen) index in species diversity is 61%.

**Table 2:** Ohalo II fish remains taxonomic composition, species richness and diversity, by studied loci (taxonomic abundance (%) is calculated according to the different taxonomic levels: Family, genus and species, and therefore the total NISP varies).

		Total		Locus 1		Locus 3		Locus 7		Locus 8	
Family	Identified Species	NISP	%	NISP	%	NISP	%	NISP	%	NISP	%
**CICHLIDAE—Total**		**3,220**	**19.0**	**728**	**6.20**	**272**	**44.2**	**1,932**	**47.0**	**288**	**53.6**
**Cichlidae**	*Oreochromis aureus*	2	0.1	0	0.00	0	0.00	2	0.29	0	0.00
**(Species level)**	*Sarotherodon galilaeus*	3	0.1	1	0.03	0	0.00	2	0.29	0	0.00
	*Coptodon zillii*	9	0.2	0	0.00	0	0.00	9	1.30	0	0.00
	*Tristramella* sp.	43	0.9	6	0.17	9	2.64	19	2.75	9	3.88
**CYPRINIDAE -Total**		**16,939**	**81.0**	**11,676**	**94.0**	**616**	**56.0**	**4,110**	**53.0**	**537**	**46.4**
**Cyprinidae**	*Luciobarbus/ Carasobarbus*	177	3.7	53	1.52	21	6.16	85	12.32	18	7.76
**(Species level)**	*Carasobarbus canis*	34	0.7	11	0.32	5	1.47	11	1.59	7	3.02
	*Luciobarbus longiceps*	74	1.5	17	0.49	10	2.93	37	5.36	10	4.31
	*Capoeta damascina*	284	6.0	78	2.24	25	7.33	131	18.99	50	21.55
	*Luciobarbus /Capoeta*	1,083	22.7	400	11.48	271	**79.47**	275	**39.86**	137	**59.05**
	*Mirogrex terraesanctae*	3,037	63.9	2,917	**83.75**	0	0.00	119	17.25	1	0.43
	**Species richness**			**6**		**4**		**8**		**5**	
	**Shannon Wiener Function**			**2.7**		**3.0**		**3.4**		**3.1**	
	**Brillouin Index (HB)**			**0.86**		**1.17**		**2.39**		**1.80**	

Species richness (S') and diversity (HB) varied among the four studied loci (Table 2). The highest values appeared in Locus 7 (S' = 8; HB = 2.4) and the lowest in Locus 3 (S' = 4; HB = 1.17).

**Fig 2:**
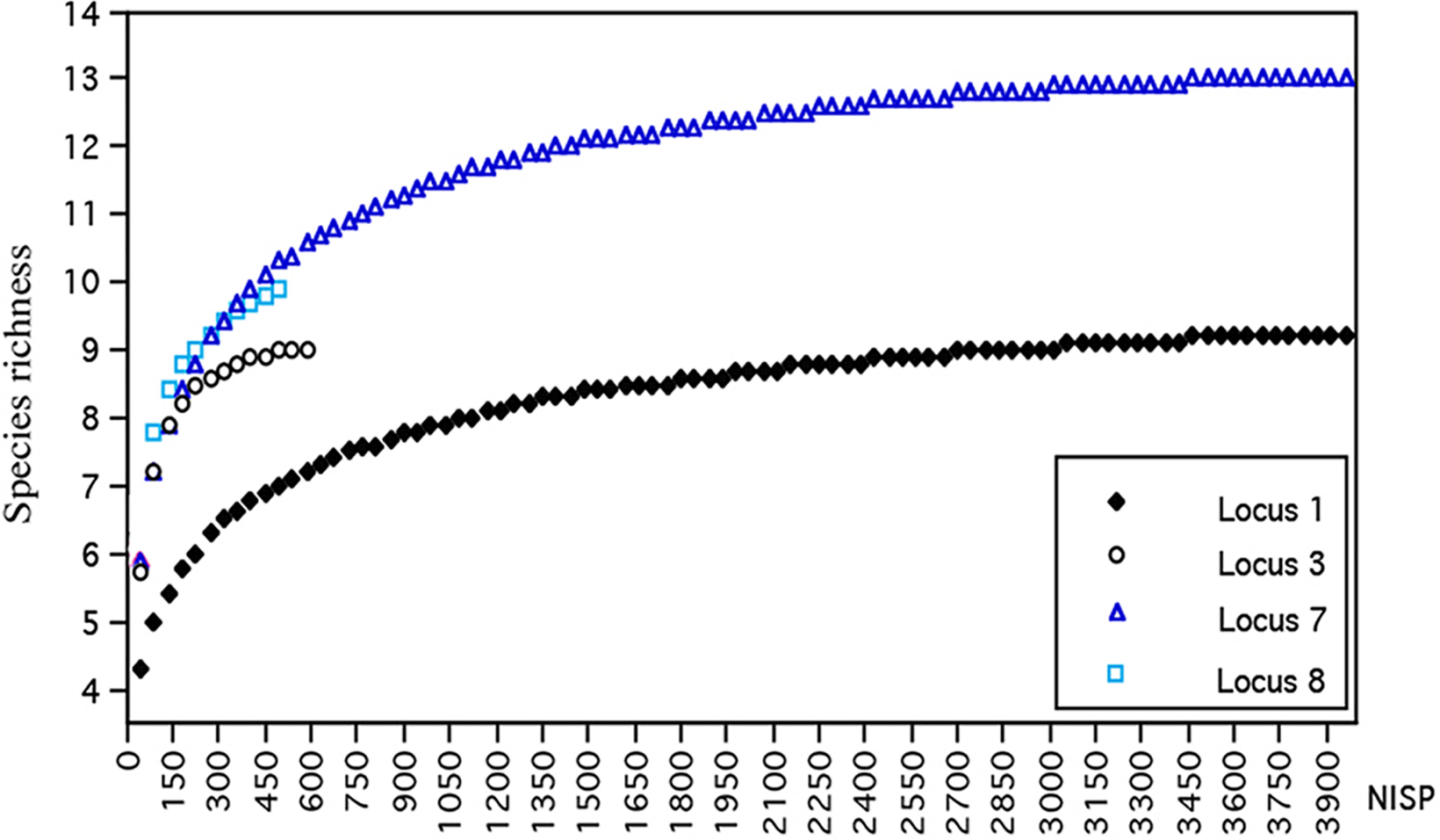
Rarefaction curves for species richness, as a function of NISP, according to the studied loci.

Rarefaction analyses indicated that the differences in species diversity among the loci would remain even after correction for sample size (Fig 2). Noteworthy, Locus 1 exhibits the highest NISP (11,676), but has a species richness (S’ = 6) lower than Locus 7, which has a much smaller sample size (NISP = 4,000; S’ = 8). Moreover, the ratio between cyprinids and cichlids (Table 2) is nearly equal (2:1) in Loci 3 (2.26:1), L.7 (2.12:1), and L.8 (1.86:1) (chi squared = 11.2, df = 2; p>.05, Table 2), while in Locus 1 it is much higher (15:1) (chi squared test = 4001.2, df = 3, p<0.0001).

Due to the relatively small number of fish identified to the species level, for further statistical analyses we grouped the remains (bones, teeth, otoliths) into four taxonomic groups: *M*. *terraesanctae*, small cyprinids, large cyprinids, and cichlids (Table 3).

**Table 3:** Total NISP and relative abundance calculated for fish recovered at Ohalo-II, according to the four taxonomic groups* and loci.

Taxonomic	Locus 1		Locus 3		Locus 7		Locus 8	
Group	NISP	%	NISP	%	NISP	%	NISP	%
*M*. *terraesanctae*	2,917	25.0	0	0.0	119	2.9	1	0.2
Small cyprinid*	7,472	64.0	12	1.9	1,520	37.0	26	4.8
Large cyprinid*	559	4.8	332	53.9	539	13.1	222	41.3
Cichlids	728	6.2	272	44.2	1,932	47.0	288	53.6
**Total NISP**	**11,676**	**100.0%**	**616**	**100.0%**	**4,110**	**100.0%**	**537**	**100.0%**

*Classification to size categories was carried out based on vertebrae centrum maximum width diameter: “small cyprinids”- < 3.5 mm; “large cyprinids” > 3.6 mm.

### Bone state of preservation

#### Skeletal element richness

The number of identified skeletal elements differed among loci. As expected, we found a high correlation between NISP and skeletal element richness (Spearman’s correlation r = .820); the highest values of skeletal element richness were in Loci 1 (S' = 74) and 7 (S' = 58). Interestingly, despite the relatively low NISP sample from Locus 8 (n = 537), skeletal element richness was relatively high (S' = 46). Table 4 presents the number of skeletal elements identified according to taxonomic group and loci. While there is no significant difference between the number of skeletal elements identified in Loci 7 and 8 (chi squared test = 1.948; df = 3; p>.05), Locus 1 significantly differs (chi squared test = 25.701; df = 6; p<0.0001; p<0.0001), probably due to the high richness of skeletal elements observed for *M*. *terraesanctae* and other small cyprinids.

**Table 4:** Number of skeletal elements identified at Ohalo II, according to the studied loci and taxonomic group.

Taxonomic group	Locus 1	Locus 3	Locus 7	Locus 8
Cichlids	30	13	35	28
Large cyprinids	48	23	40	32
*M*. *terraesanctae*	32	0	11	1
Small cyprinids	62	7	28	15

#### Skeletal element representation by anatomical region

**Fig 3:**
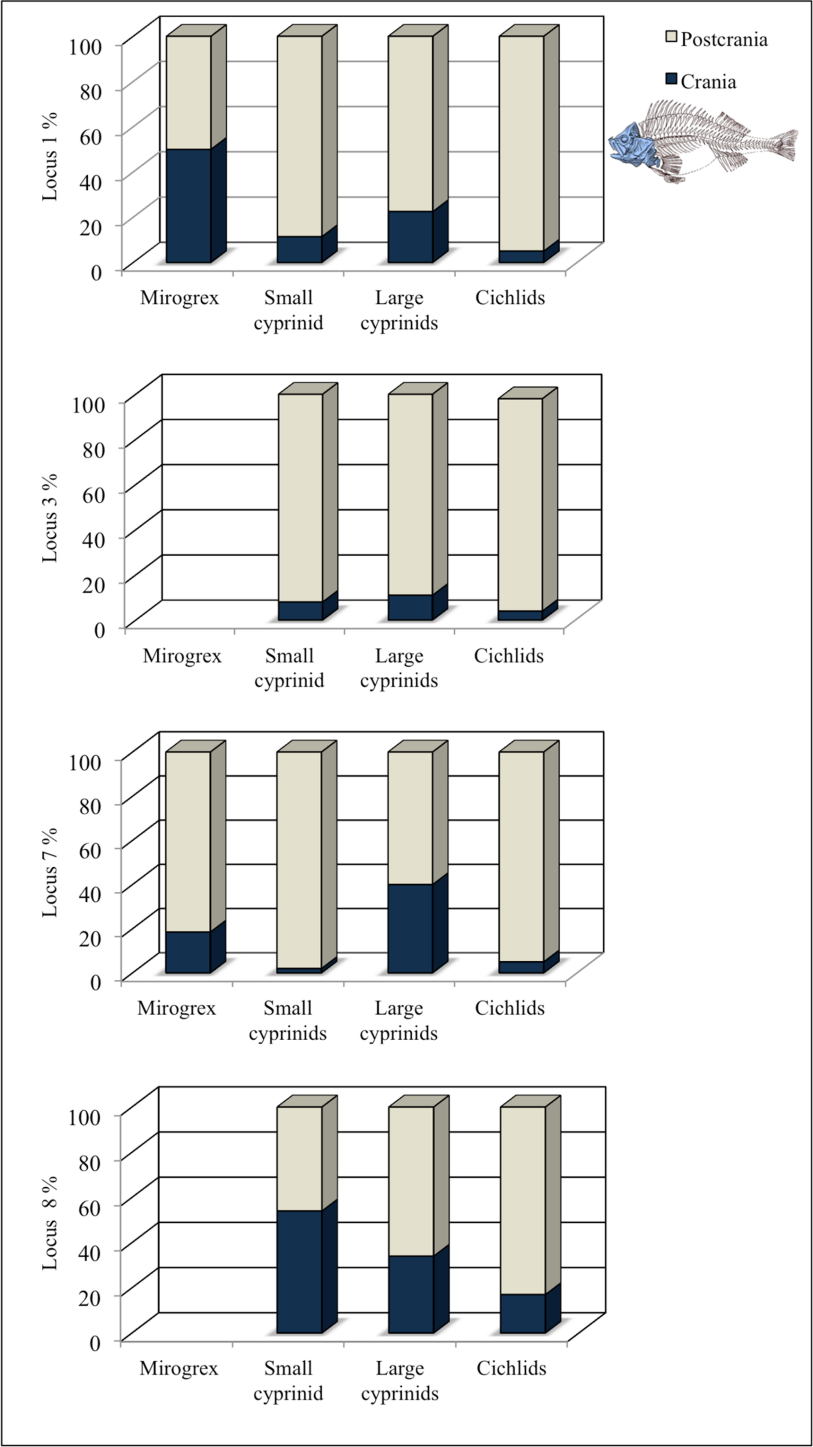
Cranial *vs*. postcranial fish remains from Ohalo II, according to taxonomic group and studied loci (fish skeleton modified from [107]).

For further analysis we grouped the bones into nine anatomical regions (Table 5), which revealed the following preservation patterns: In Locus 1, all anatomical regions are present, regardless of taxonomic group. In Loci 7 only large cyprinids and cichlids are represented by all anatomical regions. The vertebral column is significantly over-represented, regardless of excavated locus or taxonomic group: expected vertebrae frequency for the cichlid was 15%, whereas the observed frequency ranged between 60–94%; for the cyprinid the expected frequency was 18–20% whereas the observed frequency ranged between 31–93% [42]. Therefore the postcranial region is significantly over-represented at Ohalo II (survival index SI>1.3); while the cranial region is significantly under-represented for most of the taxonomic groups and excavated loci (Table 6; Fig 3). An exceptional presentation of the cranial region was observed for *M*. *terraesanctae* remains from Locus 1, for large cyprinid remains from Locus 7, and for small and large cyprinid remains from Locus 8 (Fig 3).

**Table 5:** Frequency (NISP) and percentage of skeletal elements recovered at Ohalo II, according to anatomical regions, studied loci, and taxonomic group.

		Total fish		Cyprinidae						Cichlidae	
Locus	Anatomic region			*Mirogrex*		Small		Large			
		NISP	%	NISP	%	NISP	%	NISP	%	NISP	%
**Locus 1**	**Cranial**										
	Neurocranium	713	6.1	186	6.4	507	6.8	7	1.2	13	1.8
	Branchial region	703	6.0	425	14.8	194	2.6	80	14.3	4	0.6
	Hyoid region	536	4.6	456	15.6	66	0.9	8	1.4	6	0.8
	Oromandibular region	338	2.9	252	8.6	48	0.6	29	5.2	9	1.2
	Opercular series	187	1.6	146	5.0	35	0.5	2	0.4	4	0.6
	**Postcranial**										
	Appendicular skeleton	685	5.9	129	4.4	538	7.2	9	1.6	9	1.2
	Median fins	635	5.4	46	1.6	555	6.9	21	3.8	51	7.0
	Weberian apparatus	460	3.9	89	3.0	351	4.7	20	3.6	-	-
	Vertebral column	7,419	63.5	1,188	40.7	5,216	70.0	383	68.5	632	86.8
	**Total**	**11,676**	**100%**	**2,917**	**100%**	**7,472**	**100%**	**559**	**100%**	**728**	**100%**
**Locus 3**	**Cranial**										
	Neurocranium	7	0.74	0	0.0	0	0.0	0	0.0	7	2.6
	Branchial region	30	3.16	0	0.0	0	0.0	29	8.7	1	0.4
	Hyoid region	4	0.42	0	0.0	1	8.33	3	0.9	0	0.0
	Oromandibular region	**8**	0.84	0	0.0	0	0.0	6	1.8	2	0.7
	**Postcranial**			0	0.0						
	Postcranial bones	3	0.32	0	0.0	0	0.0	1	0.3	2	0.7
	Median fins	24	2.53	0	0.0	2	16.7	17	5.1	5	1.8
	Weberian apparatus	4	0.42	0	0.0	1	8.3	3	0.9	0	0.0
	Vertebral column	868	91.56	0	0.0	8	66.7	273	82.2	255	93.7
	**Total**	**948**	**100%**	**0**	**0.0**	**12**	**100%**	**332**	**100%**	**272**	**100%**
**Locus 7**	**Cranial**										
	Neurocranium	23	0.5	1	0.8	3	0.2	3	0.6	16	0.8
	Branchial region	203	4.9	21	17.7	23	1.5	149	27.6	10	0.5
	Hyoid region	35	0.9	0	0.0	1	0.1	15	2.8	19	1.0
	Oromandibular region	82	2.0	0	0.0	4	0.3	47	8.7	31	1.6
	Opercular series	19	0.5	0	0.0	0	0.0	2	0.4	17	0.9
	**Postcranial**										
	Appendicular skeleton	92	2.2	1	0.8	7	0.5	13	2.4	71	3.7
	Median fins	648	15.8	1	0.8	54	3.5	63	11.7	530	27.4
	Weberian apparatus	17	0.4	1	0.8	10	0.7	6	1.1	-	-
	Vertebral column	2,991	72.8	94	79.0	1,418	93.3	241	44.7	1,238	64.1
	**Total**	**4,110**	**100%**	**119**	**100%**	**1,520**	**100%**	**539**	**100%**	**1,932**	**100%**
**Locus 8**	**Cranial**										
	Neurocranium	12	2.2	0	0.0	2	8.0	0	0.0	10	3.5
	Branchial region	59	11.0	0	0.0	3	12.0	46	20.7	10	3.5
	Hyoid region	9	1.7	0	0.0	0	0.0	5	2.3	4	1.4
	Oromandibular region	40	7.5	0	0.0	5	19.0	23	10.4	12	4.2
	Opercular series	20	3.7	0	0.0	4	15.0	2	0.9	14	4.9
	**Postcranial**				0.0						
	Appendicular skeleton	33	6.2	0	0.0	0	0.0	13	5.9	20	6.9
	Median fins	69	12.9	0	0.0	3	11.0	25	11.3	41	14.2
	Weberian apparatus	7	1.3	0	0.0	1	4.0	6	2.7	-	-
	Vertebral column	288	53.6	1	100.0	8	31.0	102	46.0	177	61.5
	**Total**	**537**	**100%**	**1**	**100%**	**26**	**100%**	**222**	**100%**	**288**	**100%**

**Table 6:** Frequency (NISP), percentage, and survival index (SI) calculated for cranial and postcranial bones, according to the studied loci at Ohalo II.

Excavated	Taxonomic	Cranial region			Postcranial region			Total
Area	group	NISP	%	SI	NISP	%	SI	NISP
**Locus 1**	*M*. *terraesanctae*	1,465	50.2	0.76*	1,452	49.8	1.46*	2,917
	Small cyprinids	850	11.4	0.18*	6,622	88.6	2.39*	7472
	Large cyprinids	126	22.5	0.36*	433	77.5	2.09*	559
	Cichlids	36	4.9	0.08*	692	95.1	2.57*	728
**Locus 3**	*M*. *terraesanctae*	0	0.0	0.00	0	0.0	0.00	0
	Small cyprinids	1	8.3	0.84*	11	91.7	5.39*	12
	Large cyprinids	38	11.4	0.05*	294	88.5	3.54*	332
	Cichlids	10	3.7	0.02*	262	96.3	4.38*	272
**Locus 7**	*M*. *terraesanctae*	22	18.5	0.28*	97	81.5	2.40*	119
	Small cyprinids	31	2.0	0.03*	1,489	98.0	2.65*	1,520
	Large cyprinids	216	40.1	0.64*	323	59.9	1.62*	539
	Cichlids	83	4.8	0.08*	1,839	95.2	1.51*	1,932
**Locus 8**	Small cyprinids	14	54.0	0.850	12	46.0	1.25	26
	Large cyprinids	76	34.2	0.54*	146	65.8	1.78*	222
	Cichlids	50	17.4	0.28*	238	82.6	1.31*	288

*Chi-squared test: significantly different from the expected value p<0.001.

#### Bone preservation and fragmentation

**Fig 4:**
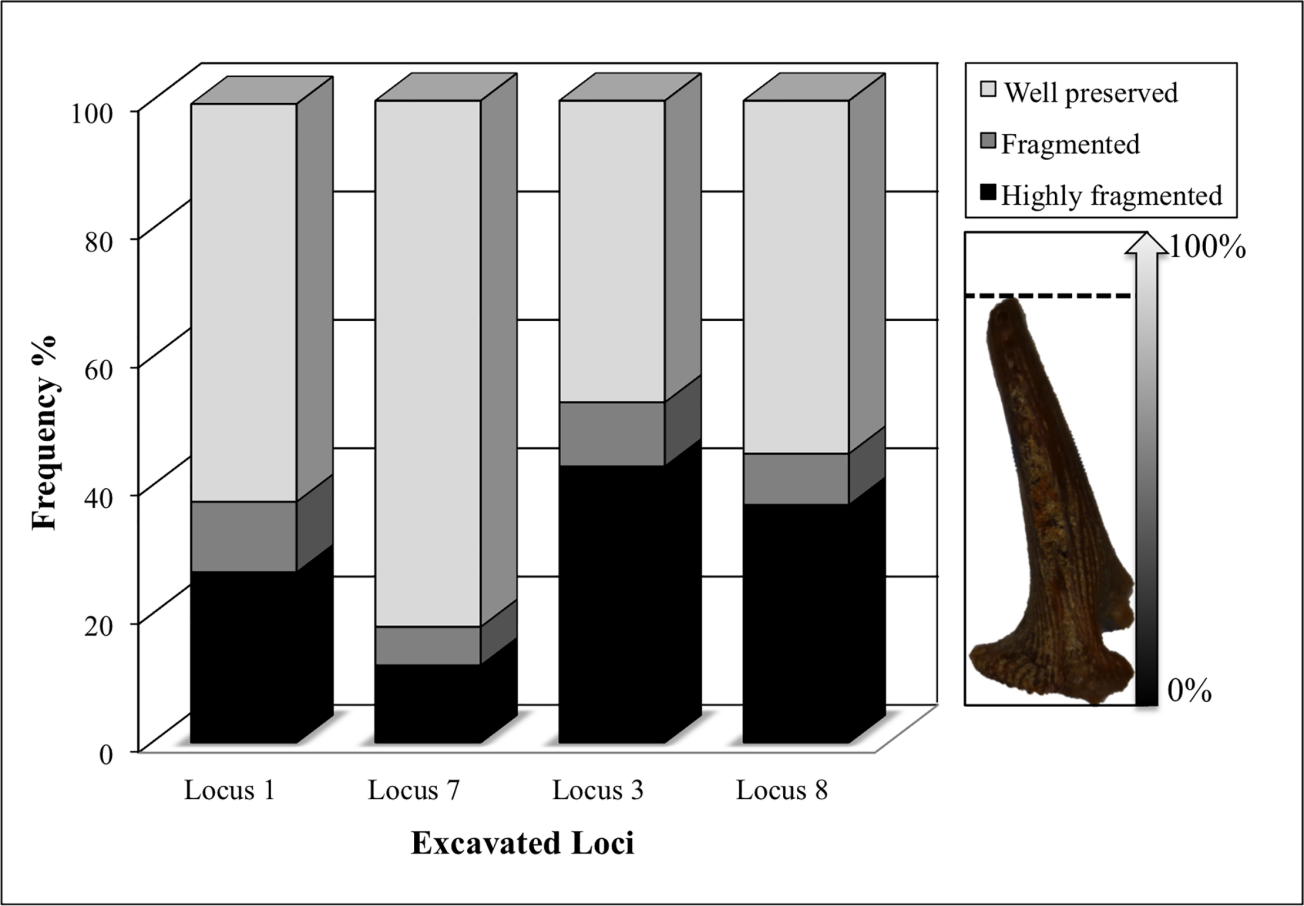
State of bone preservation at Ohalo II, according to studied loci (chi squared test = 944.133; p = 0.0001; df = 6).

We found a significant difference in bone preservation and fragmentation patterns among loci (chi squared = 944.133; df = 6; p = 0.0001). The bones from Locus 7 were best preserved, followed by Locus 1 (Fig 4). The bones from Loci 3 and 8 exhibited a high degree of fragmentation. The best-preserved skeletal elements in all loci were the vertebrae. Bones from the oromandibular region and opercular apparatus were highly fragmented in large cyprinids and cichlids. We did not observe any difference in state of fragmentation between the cranial and postcranial regions.

#### Signs of burning (bone color)

Most of the bones (87%) displayed brown and dark brown colors (Table 7), with a small sample (ca. 9%) displaying various colors, ranging from black to white. Examination of the mineralogical content with FTIR revealed that bones displaying brown, dark-brown and black colors were mineral-stained (ca. 20%); bones with gray and white colors were burned (ca. 6%), and therefore indicative of human impact; and bones with orange-brown color (ca. 3%) were oxidized, suggesting that some of the fish remains had been periodically exposed to the open air (probably during periods when the lake’s water level was low). Based on the FTIR analysis, signs of burning were identified in ca. 12% of the remains recovered at Locus 7 (ashes). In Locus 1, less than 1% of the fish remains exhibited signs of burning.

**Table 7:** Frequency (NISP) of colors and signs of burning recorded on fish remains from Ohalo II, according to the studied loci.

	Locus 1		Locus 3		Locus 7		Locus 8		Total	
Bone Color	NISP	%	NISP	%	NISP	%	NISP	%	NISP	%
**Black**	244	2.9	65	7.0	293	5.4	22	4.0	624	4.1
**Gray**	42	0.5	92	10.0	493	9.2	11	2.0	638	4.2
**White**	35	0.4	15	1.6	152	2.82	3	0.5	205	1.3
**Dark brown**	1,124	13.2	227	24.6	1,014	18.8	148	26.6	2,513	16.4
**Brown**	6,942	81.8	498	54.0	3,078	57.2	343	61.6	10,861	70.7
**Light Brown**	44	0.5	1	0.1	20	0.4	2	0.4	67	0.4
**Orange-Brown**	59	0.7	25	2.7	334	6.2	28	5.0	446	2.9
**Total**	**8,490**	**100.0%**	**923**	**100.0%**	**5,384**	**100.0%**	**557**	**100.0%**	**15,354**	**100.0%**

### Fish bone spatial distribution

In all the studied loci, the fish remains exhibited a clumped distribution pattern with a high value of bone mean scatter frequency (BSF) (range of 90–930 bones per 0.25 m^2^).

#### Locus 1

**Fig 5:**
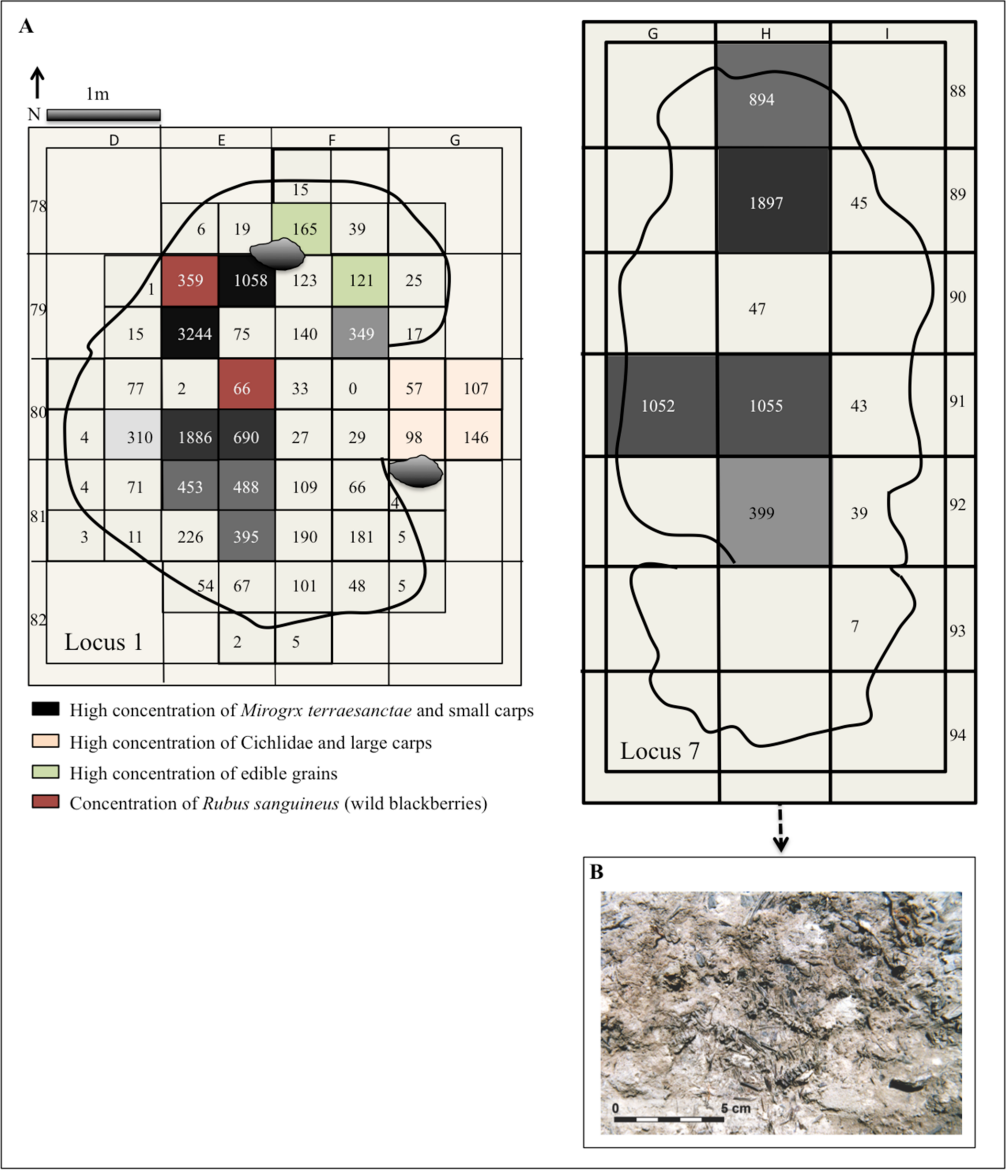
A. Fish remains spatial distribution pattern in Loci 1 (floors I and II combined) and 7 (in Locus 1 areas with a large concentration of edible grains from floor II are marked, after [40]; B. In-situ fish skeletons recovered at Locus 7.

The 11,676 fish remains sampled from 53 squares (square = 50X50X0.05 cm) exhibited a concentrated and clumped distribution pattern (Id = 5.65, MU = 0.99, MC = 1.01, Ip = 0.60), with an average BSF value of 930 bones per 0.25 m^2^ (Fig 5A). Most of the remains were recovered from Floor II (82%), and only 16% from Floor I. On Floor I most of the remains were concentrated in two sub-squares: E80d (36%) and E81d (20%); and on Floor II most were concentrated in three sub-squares: E79 b (11%), E79c (35%) and E80c (20%) (Fig 5A). The concentration pattern of the fish bones greatly varied (up to 50-fold) in different parts of the structure. Remains of large cyprinids and cichlids were recovered in the eastern part of the hut, mainly in square G 80. Small carp bones were abundant in squares E 79–80, located in the western part of the hut (Fig 5A).

#### Locus 3

The 616 fish remains sampled from this structure exhibited a concentrated and clumped distribution (Id = 2.0606, MU = 0.9933, MC = 1.0106, Ip = 0.5584) with a BSF average value of 190 bones per 0.25 m^2^.

#### Locus 7

The 4,110 fish remains sampled from ten squares exhibited a dense concentration and an average BSF of 843 bones per 0.25 m^2^ (Fig 5). Some of the vertebral columns were intact, representing deposition of complete fish (Fig 5B). The squares with the highest concentration of fish remains were located in two areas: in the center (G-H 91) and in the northern section (H 88–89) of the locus.

#### Locus 8

The 537 fish remains sampled from this locus were concentrated in two sub-squares, and therefore were clearly clumped in their distribution pattern.

### Fish economic value

The fish exploitation index (FEI) calculated for each locus revealed the economic value of large fish (*L*. *longiceps*, *C*. *canis*, *C*. *damascina* and cichlids) to the inhabitants of Ohalo II. In Loci 3, 7, and 8, large fish dominated the assemblage (FEI for Locus 3 = .98, Locus 7 = .60, Locus 8 = .95), while in Locus 1 large fish were rare (FEI = .11), with a preponderance of small-sized cyprinids and *M*. *terraesanctae* (Kinneret Bleak).

### Natural vs. Cultural accumulation

The characteristics of our studied natural death assemblage (located ca. 150 meter north-west of Ohalo II site) and those of Ohalo II assemblages are given in Table 8. While the fish assemblage excavated at Locus 1 shows similar characteristics to those of the natural death assemblage, those of Loci 3, 7, and 8 differ in three major aspects: abundance of large carps and cichlids, absence or rarity of Kinneret bleak and small cyprinids, and burning marks on ca. 10% of the bones.

**Table 8:** Comparison between diagnostic criteria of naturally accumulated fish remains vs. fish remains recovered at Ohalo II (OH), according to the studied loci (loci 1, 3, 7, 8).

Diagnostic Criteria	Natural assemblage	OH- Locus 1	OH-Locus 3	OH-Locus 7	OH-Locus 8
**Sample size NISP**	5,037	11,676	616	4,110	537
**Color and dispersion:**					
**Mean bone scatter frequency (BSF)**	423 bones per 0.25sqm (range 8–2840 bones)	930 bones per 0.25sqm	190 bones per 0.25sqm	842 bones per 0.25sqm	Not enough data
**Morisita Index of dispersion-Id**	6.52	5.65	2.06	2.25	-
**Bone dispersion pattern**	Clumped	Clumped	Clumped	Clumped	Clumped
**Color +burning signs**	Brown (light to dark) no burning signs	> 99% brown 1% <burning signs	90% Brown, 10% burnt	88% Brown, 12% burnt	97.5% Brown, 2.5% burnt
***Taxonomic composition*:**					
**Highest taxonomic richness**	S = 5	S = 6	S = 4	S = 8	S = 5
**Highest taxonomic diversity (Brillouin's Index)**	HB = 1.59	HB = 0.86	HB = 1.17	HB = 2.4	HB = 1.8
**Family representation**	Cyprinidae 80% Cichlidae 6% Clariidae 6%-only on surface layer	Cyprinidae 90% Cichlidae 6%	Cyprinidae 55% Cichlidae 44%	Cyprinidae 53% Cichlidae 47%	Cyprinidae 46% Cichlidae 54%
**Abundant taxa**	*M*. *terraesanctae*, small cyprinids	*M*. *terraesanctae*, small cyprinids	Large cyprinids& cichlids	Large cyprinids& cichlids	Large cyprinids& cichlids
**Fish Exploitation Index**	0.182	0.11	0.98	0.60	0.95
***Skeletal representation*:**					
**Skeleton richness**	55	74	30	58	46
**Scales**	Clumps of scales in all taphofacies.	No scales	No scales	No scales	No scales
**Otoliths**	No otoliths	No otoliths	No otoliths	No otoliths	Cichlids otoliths
**Crania vs. postcrania:**					
***M*. *terraesanctae***	Crania region over represented	Crania region well preserved but under- represented	No cranial bones	No cranial bones	No cranial bones
**Large cyprinids**	Crania region over represented	Crania region under-represented	Crania region under-represented	Crania region under-represented	Crania region under-represented
**Cichlids**	Crania region under-represented	Crania region under-represented	Crania region under-represented	Crania region under-represented	Crania region under-represented
**Vertebral column SI**	Over-represented for all taxa in all samples	Over-represented for all taxa	Over-represented for all taxa	Over-represented for all taxa	Over-represented for all taxa

**Fig 6:**
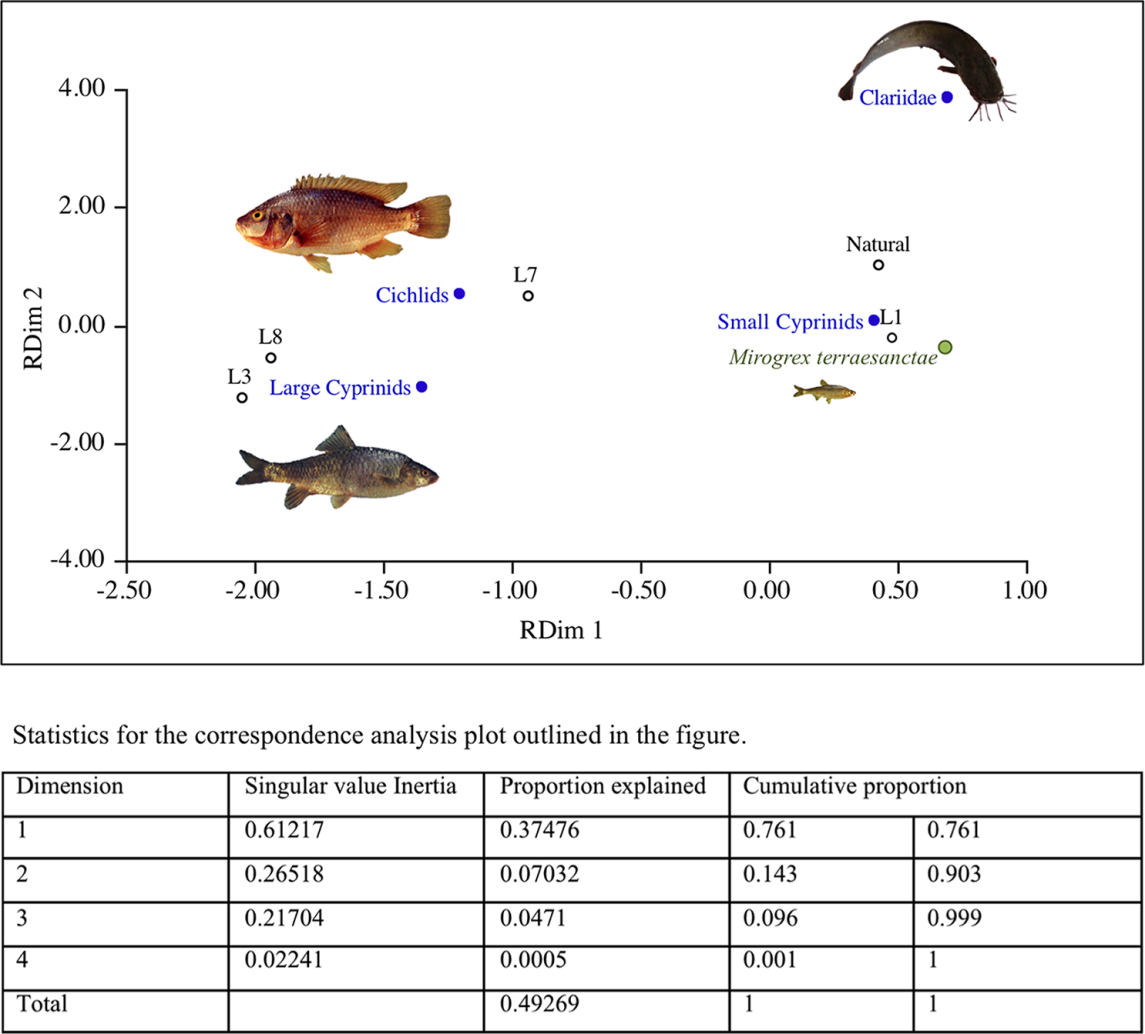
Correspondence analysis of taxonomic groups’ relative abundance (%) in the natural accumulation and at Loci 1, 3, 7, and 8.

MDS analysis clustered Loci 3, 7, and 8 assemblages together, and distinctively separate from the natural accumulation (Fig 6), implying a similarity in fish pattern of exploitation (e.g., a preference for cichlids and large carps). The Locus 1 fish assemblage, in contrast, exhibited a similarity with the natural accumulation, mainly due to the high presence of Kinneret bleak at both places.

## Discussion

Recent zooarchaeological and isotopic studies have indicated a sharp shift in the role of aquatic resources in the diet and economy of ancient populations since the mid-Upper Paleolithic [1, 5, 108–110]. The present study provides solid data indicating that the diet breadth during the Upper Paleolithic was broader than has been previously assumed, and that freshwater fish were an important dietary component.

The incorrect notion that fish played only a small role in the Late Pleistocene human diet is probably due to the rarity of systematic studies on fish remains and fishing among prehistoric populations [15, 17], as well as preservation bias, as witnessed from ethnographic studies [71, 111–114]. As a result, fish have been portrayed as an unusual exception and thus as an unimportant dietary element [1, 17, 115]. The special taphonomic conditions at Ohalo II, i.e., a water-logged site covered by clay [102, 116], immediately following its abandonment (as attested by the variety of fragile *in-situ* remains on the floors), have resulted in excellent preservation of the fish remains, providing a rare opportunity to examine fish exploitation at the end of the Upper Paleolithic.

Prior to any discussion of human fishing activities at any water-logged site, it is important to examine the possibility that the fish bones could have accumulated at the site due to natural death of the fish rather than as a result of human activity. The comparison of Ohalo II fish bone assemblages from Loci 3, 7, and 8 with the natural death assemblage, excavated beyond the range of the Ohalo II site [74] (Table 8), revealed a number of differences that attest to fishing activity at the site. For example, maximum species richness was higher at Ohalo II (S = 8) than at the natural death assemblage site (S = 5); the diversity index of the death assemblage was significantly lower (HB = 0.38–1.594) compared to Loci 3, 7, and 8 (HB = 1.17–2.398); the natural death fish exploitation index was much lower (0.1) relative to the indices calculated for Ohalo II (>0.6), probably due to the abundance of large carps and cichlids in these loci; and the natural death assemblage was characterized by over-representation of the cranial region (of *M*. *terraesanctae*, large cyprinids and catfish), whereas at the Ohalo II loci they were under-represented. Exceptional in the comparison was the clumped distribution pattern with a high NISP for bone-scatter frequency, which was expected to differ between the two assemblages [71, 73, 85, 86], but did not. Unlike Loci 3, 7, and 8, Locus 1, which displayed an exceptional state of preservation, showed a high preponderance of *M*. *terraesanctae* and small cyprinids, similar to that of the natural death assemblage. The cultural and taphonomic implications of this finding are discussed separately in section 5.2B.

### Does Ohalo II display the characteristics of an artisanal fishing community?

Ethnographic and ethno-archaeological studies have demonstrated that traditional fishing sites possess a number of common physical characteristics (Table 9) [71, 73, 112, 117–121]. We examined these characteristics (Table 9) in order to gain further understanding of the role of fish and fishing at Ohalo II.

**Table 9:** Characteristics of traditional fishing camps based on ethnographic data, compared to Ohalo II characteristics (following [42, 73, 120, 122–125].

Feature	Traditional fishing camp	Ohalo II
**Location**	Within the vicinity of the shoreline (ca. 100 m)	Within the vicinity of the shoreline
**Size**	200–7,000 sqm	Ca. 2,000 sqm
**Constructions**	Semi-circular structures with hearths	Oval structures, some with hearths
**Fish production area**	Outside of the dwelling huts	Locus 8 may attest to a specific area for fish long-term preservation
**Fishing tools**	Weirs, baskets, rakes, wooden traps, cretated barriers, hands, gill nets, cast nets, ichthyotoxic plants, harpoons, bow and arrow, hooks and lines etc.	Double-notched weights, charred cord, flint microliths
Animal remains*	Mammals, reptiles, and birds	Mammals, reptiles, and birds
**Length of stay**	Seasonal repeated occupations for short or long term	Repeated occupations for short or long term, during different seasons.
**Tools for food processing**	Pestles and mortars	Large and shallow stone bowls

*Mollusks are not included as it is impossible to differentiate in waterlogged site between natural and cultural assemblages.

### A. Site features

Data on artisanal small-scale fishing groups indicate that the majority of present-day fishing camps are located within the vicinity of the shoreline (ca. 100 m), vary in size according to length of stay (200–7000 sqm), and include several semi-circular structures [73, 126] similar to what has been observed at Ohalo II [36, 37]. At Lake Turkana, for example, the structures serve as sleeping shelters (windbreaks) and/or to protect the hearths inside. Hearth(s) and grind-stones, commonly found within these traditional structures, were also found within the structures at Ohalo II [18, 127–129].

Many of the fishing camps have been repeatedly occupied (for example 60% at Lake Turkana) [42, 85, 130], and include in addition to fish remains, bones of reptiles, mammals, and birds, [73], as also reported for Ohalo II site [23, 24, 34, 131]. This attests to the diverse subsistence economy of artisanal fishing villages. The data presented in Table 9, lend further support, though from a different angle, that Ohalo II presents characteristics similar to those of a present-day artisanal fishing village, and that the inhabitants were engaged in intensive aquatic activities throughout the year, by the same cultural entity.

No less important is the observation from ethnographical studies that fish preservation en masse does not require either specially constructed facilities or salt, or a commercial motive [66, 114, 117]. The Lake Turkana fishing camps have no constructed facilities for long-term preservation, and preservation activities usually take place outside of the dwelling huts. Ohalo II may present a similar situation. The large number of well-preserved fish remains (crania and post-crania) recovered in a small pit (Locus 8) outside of the dwelling huts may provide further evidence of fish preservation at Ohalo II. If this is the case, then Ohalo II offers the earliest evidence of long-term fish preservation in the southern Levant.

Cultural artifacts directly associated with fishing are usually rare at prehistoric sites, hindering our understanding of past fishing technology and efficiency [117]. Nevertheless, several artifacts that have been retrieved from Ohalo II may shed light on past local fishing techniques. The presence of double-notched pebbles and small pieces of charred cord may indicate the use of fishing nets or weirs [116, 127, 132–134]. Flint microliths and small bone tools identified at the site [23, 135] could have been employed for hook and line fishing. Finally, considering fish breeding behavior, hand fishing could also have been used (Table 1).

### B. Characteristics of the fish remains

In the previous sections we have demonstrated that Ohalo II manifests many of the site characteristics of a small-scale fishing community and that the characteristics of the various fish assemblages retrieved at the site differ from those of the natural death fish bone assemblages. In this section we compare the Ohalo II fish assemblages with assemblages uncovered at other fishing sites.

*Taxonomic diversity*: The Ohalo II fish assemblages are characterized by low taxonomic diversity (Sørensen index 61%), i.e., only two families out of seven (Cyprinidae and Cichlidae) were present at the site. This finding is in line with other studies showing that fish assemblages derived from targeted exploited species of littoral/shallow water manifest a low taxonomic diversity (they do not include non-edible species) [13, 73, 114, 117, 136].

#### Skeletal representation

In traditional fishing camps, skeletal element representation largely depends on the type of processing activities carried out at the site [8, 66, 69, 111, 112, 114, 119]. For example, at fish production sites vertebral elements are rare or totally absent (as they are transported away from the site). This is in contrast to base camps, where vertebral remains are over-represented relative to cranial remains [13, 114, 137]. Moreover, culinary practices and fish cooking reduce survivorship of fish remains, and may therefore contribute to a biased skeletal representation [68, 114, 138, 139]. At Ohalo II we have identified, for the first time, all of the characteristics of a final Upper Paleolithic long-term fishing camp where fish were intensively exploited and preserved.

#### Cut marks

The fact that no cut marks were identified on the fish bones from Ohalo II is not surprising since, in general, prior to the use of metal knives cut marks on fish bones are generally faint, small, difficult to identify, and tend to dissolve through time [13, 73, 114, 140, 141]. For example, at the coastal site of Tel Dor, dated to the Iron Age, out of 756 fish bones analyzed only five cranial bones of *Lates niloticus* (ca. 0.6%) presented cut marks [142].

#### Burning signs

In present-day fishing villages changes in bone color due to fish roasting appear on 3–20% of the remains [114]. At Ohalo II, clear evidence of burning (gray/white color) was identified on ca. 12% of the fish bones. Experimental studies have shown that a gray/white color is reached when fire temperature is over 500°C [68, 87, 88, 138]. Consequently, these bones have a lower chance of survival [68]. It is interesting that most of the bones displaying signs of burning were from the hearth of Locus 7. FTIR analyses revealed that the dark-colored bones are a result of natural mineral staining and not of exposure to fire [42, 143].

### Can we identify evidence of school and pelagic fishing at Ohalo II?

The Ohalo II fish remains exhibit two distinctive patterns of exploitation: 1) a dominance of large cyprinids and cichlids in three of the studied loci; and 2) a dominance of small cyprinids, especially the Kinneret bleak (*Mirogrex terraesanctae*) in Locus 1. The preponderance of small cyprinids, mainly *M*. *terraesanctae*, in Locus 1 raises the question: Were *M*. *terraesanctae* exploited by the Ohalo II inhabitants, and if so, how were they caught and what was their role in the diet of the local inhabitants? To address this question, we first need to discuss *M*. *terraesanctae*'s ecology, economic importance, fishing techniques, and depositional history [74].

#### The Kinneret bleak–*Mirogrex terraesanctae*

The Kinneret bleak (*M*. *terraesanctae*) is a small pelagic fish (total length 220 mm) endemic to Lake Kinneret [144, 145]. Although pelagic, during the breeding season, from November to May with a peak in mid-winter (January-February), it spawns in the shallow littoral zone (0–50 cm). Spawning begins shortly after nightfall, when schools of fish move along the shoreline in rocky regions and release their milt and eggs. The adhesive eggs attach to the surface of recently inundated, algae-free stones [144]. *M*. *terraesanctae* is currently highly abundant in Lake Kinneret and constitutes more than 50% of the annual commercial catch [146, 147]. However, despite its abundance, it is currently regarded as a tasteless fish and of low economic value [147].

#### *Mirogrex terraesanctae*: Cultural exploitation or natural death?

Assuming that the *M*. *terraesanctae* remains recovered at Locus 1 represent human activity, then this is the first and earliest evidence of the mass harvesting of small pelagic fish, in the Levant, either from the pelagic region or from the littoral zone during the fish-breeding season (winter). Either of these two options is unique and would constitute the earliest evidence of either night fishing of targeted small-sized fish or of pelagic fishing. If Ohalo II inhabitants were indeed engaged in any of these exceptional activities, then among the four studied loci, it is currently evident only at Locus 1 (a unique structure in many other archaeological aspects [27, 40, 134]). Moreover, if such exceptional fishing technology and activity indeed existed at Ohalo II, it then disappeared from the known Levantine archaeological records and reappeared only in later historic periods [15, 17].

There are two main arguments, however, that speak against the possibility of school fishing at Ohalo II: 1). Today *M*. *terraesanctae* is considered as a tasteless species and of low economic value; and 2). *M*. *terraesanctae* assemblage characteristics in Locus 1 bear a similarity to those of the mass death natural assemblage in the lake’s clay sediment (Table 8) [74]. A natural death assemblage with a high percentage of *Mirogres* sp. was also identified from Lake Hula [16, 77]

Moreover, a comparison between the fish remains’ spatial distribution and activity areas identified at Locus 1 (Floor II) reveals that the clumped dispersal pattern observed for *M*. *terraesanctae* and small cyprinids (Fig 5A) differs from that observed for the large fish, lithics, and edible plant remains recovered in association with floor II [25, 38, 40, 134]. While this pattern may shed further information in regard to different areas of activities in Locus 1, an examination of the wetland botanical remains reveals their preponderance in Locus 1, and in the vicinity of *M*. *terraesanctae* remains [40, 134]. For example, the exceptional remains of unburned *Rubus sanguineus* (wild berry) were recovered in the same square E79a, as *M*. *terraesanctae* [40]. Since these berries are usually consumed immediately, and do not preserve well in archaeological assemblages, Weiss et al., (2008) could not explain these finds. From an environmental and ecological perspective *Rubus sanguineus* may also represent the natural wetland vegetation that grew during periods of high water level [52, 102, 134].

#### Was fishing a year-round activity or carried out sporadically at Ohalo II?

Studies have revealed that for the last 40,000 years ancient populations have sporadically exploited fish, which provide a high-return meal as they are easy to catch due to their breeding behavior and migration routes [1, 9, 15, 72, 85, 148–151]. During the Upper Paleolithic, for example, Clariidae (catfish) were among the most heavily exploited fish in Africa and Egypt [152–155], and Salmonidae in Europe and North America [1, 65, 137, 156–160]. The reproduction behavior of these fish and the shallow habitat in which they breed make them extremely vulnerable to fishing.

The fish exploitation patterns observed at Ohalo II accord with those observed at other Upper Paleolithic sites. The diversity of fish (Tables 1 and 2) indicates that their exploitation pattern was based on seasonal abundance and breeding behavior (in the littoral zone of Lake Kinneret and nearby rivers) (Table 1). The cichlids breed along the shore in spring and summer (April-September) [52], while the cyprinids breed in winter (January-April), in running streams and along the littoral zone. This suggests that fishing was part of the daily-life activities of the Ohalo II inhabitants and that it was not sporadic and opportunistic activity but rather, practiced routinely throughout most of the year. The Kinneret fish breeding locations facilitated littoral fishing and ensured a constant supply of high-return, low-cost food for the Ohalo II people. A large subsistence return for minimal procurement efforts has also been documented for traditional fishing communities [8, 9, 85, 108, 114, 161], and was argued to be the major cause of population concentration for an extended period of time in a limited area of wetland habitat [134].

## Summary

Upper Paleolithic economies are characterized by a sharp increase in the exploitation of diverse small-sized animals [2, 4–6, 162–164]. Although fish are listed among these low-ranked species, to the best of our knowledge no large-scale study has been conducted on the role of fish during this period [15, 115]. The role of fish exploitation may consequently have been largely underestimated, with fishing having been incorrectly attributed to periods of economic stress following a dearth of the more traditional food items.

The findings from the current study of the fish remains from Ohalo II greatly change this view and indicate that aquatic habitats too played an important role in the diet and economy of past populations, greatly contributing to their stability [15, 16, 86, 115, 134, 165]. The Ohalo II fish remains offer the sole and earliest evidence to date of a fisher-hunter-gatherer economy along the Lake Kinneret shores, immediately after the peak of the Last Glacial Maximum [15, 17–19, 22, 26]. This economy reveals a complex taphonomic scenario representing evidence of fish preparation and consumption, as part of a very rich diet that encompassed a variety of mammals and birds and, no less important, a wide range of plant foods, including cereals, of which some may have been cultivated [39, 166, 167].

## Supporting information

S1 Table Data (xls file) with Ohalo II fish remains and specimen catalogue numbers. (XLS)
